# Supplementary material for: Harmonizing and integrating the NCI Genomic Data Commons through accessible, interactive, and cloud-enabled workflows
Source: PLoS One. 2025 Mar 4;20(3):e0318676. doi: 10.1371/journal.pone.0318676 (PMC11878898; doi:10.1371/journal.pone.0318676)
Supplement: S1 File — (PDF) [file pone.0318676.s001.pdf]

Ensembl gene ID	gene symbol	GDC v15 counts	GDC v32 counts	Summary from GeneCards or NCBI Gene
ENSG00000157654	PALM2AKAP2	7	2536	This gene belongs to the paralemmin downstream gene (PDG) family defined in PMID:22855693. Paralemmin downstream genes may have evolved contiguously with the paralemmin genes and are associated with other paralemmin paralogs in humans and several other taxa. The gene encodes three distinct protein isoforms, the PALM2 isoform, the AKAP2 isoform and the PALM2-AKAP2 isoform. The biological significance of the PALM2-AKAP2 isoforms is yet unknown. Earlier, PALM2 and AKAP2 were annotated as separate genes and PALM2-AKAP2 was annotated as a readthrough gene. [provided by RefSeq, May 2019]
ENSG00000197753	LHFPL5	2	659	This gene is a member of the lipoma HMGIC fusion partner (LHFP) gene family, which is a subset of the superfamily of tetraspan transmembrane protein encoding genes. Mutations in this gene result in deafness in humans, and a mutation in a similar gene in mice results in deafness and vestibular dysfunction with severe degeneration of the organ of Corti. It is proposed to function in hair bundle morphogenesis. [provided by RefSeq, Jul 2008]
ENSG00000245864	MEF2C-AS2	1	147	MEF2C-AS2 (MEF2C Antisense RNA 2) is an RNA Gene, and is affiliated with the lncRNA class. Diseases associated with MEF2C-AS2 include Diffuse Large B-Cell Lymphoma.
ENSG00000250891	LINC02208	1	102	LINC02208 (Long Intergenic Non-Protein Coding RNA 2208) is an RNA Gene, and is affiliated with the lncRNA class
ENSG00000253194	AL137009.1	1	635	ENSG00000253194 (Novel Transcript, Antisense To FAM184A) is an RNA Gene, and is affiliated with the lncRNA class.
ENSG00000260007	AC107871.1	1	496	ENSG00000260007 (Novel Protein) is a Protein Coding gene
ENSG00000265817	FSBP	1	157	Enables identical protein binding activity. Located in nucleoplasm. [provided by Alliance of Genome Resources, Apr 2022]
ENSG00000279170	TSTD3	3	568	TSTD3 (Thiosulfate Sulfurtransferase Like Domain Containing 3) is a Protein Coding gene. Diseases associated with TSTD3 include Leber Congenital Amaurosis 19.
